# Design and Numerical Analysis of a Graphene-Coated SPR Biosensor for Rapid Detection of the Novel Coronavirus

**DOI:** 10.3390/s21103491

**Published:** 2021-05-17

**Authors:** Tarik Bin Abdul Akib, Samia Ferdous Mou, Md. Motiur Rahman, Md. Masud Rana, Md. Rabiul Islam, Ibrahim M. Mehedi, M. A. Parvez Mahmud, Abbas Z. Kouzani

**Affiliations:** 1 Department of Electrical and Electronic Engineering, Rajshahi University of Engineering and Technology, Rajshahi 6204, Bangladesh; abdulakibruhe@gmail.com (T.B.A.A.); samia.mou.bauet@gmail.com (S.F.M.); motiurrahmanbauet@gmail.com (M.M.R.); md.masud.rana.ruet@gmail.com (M.M.R.); 2 Faculty of Engineering and Information Sciences, University of Wollongong, Wollongong, NSW 2522, Australia; mrislam@uow.edu.au; 3 Department of Electrical and Computer Engineering (ECE) and Center of Excellence in Intelligent Engineering Systems (CEIES), King Abdulaziz University, Jeddah 21589, Saudi Arabia; imehedi@kau.edu.sa; 4 School of Engineering, Deakin University, Geelong, VIC 3216, Australia; abbas.kouzani@deakin.edu.au

**Keywords:** biosensor, coronavirus, COVID-19, molecular detection, rapid detection, SARS-CoV-2, sensor, spike receptor-binding domain, surface plasmon resonance

## Abstract

In this paper, a highly sensitive graphene-based multiple-layer (BK_7_/Au/PtSe_2_/Graphene) coated surface plasmon resonance (SPR) biosensor is proposed for the rapid detection of the novel Coronavirus (COVID-19). The proposed sensor was modeled on the basis of the total internal reflection (TIR) technique for real-time detection of ligand-analyte immobilization in the sensing region. The refractive index (RI) of the sensing region is changed due to the interaction of different concentrations of the ligand-analyte, thus impacting surface plasmon polaritons (SPPs) excitation of the multi-layer sensor interface. The performance of the proposed sensor was numerically investigated by using the transfer matrix method (TMM) and the finite-difference time-domain (FDTD) method. The proposed SPR biosensor provides fast and accurate early-stage diagnosis of the COVID-19 virus, which is crucial in limiting the spread of the pandemic. In addition, the performance of the proposed sensor was investigated numerically with different ligand-analytes: (i) the monoclonal antibodies (mAbs) as ligand and the COVID-19 virus spike receptor-binding domain (RBD) as analyte, (ii) the virus spike RBD as ligand and the virus anti-spike protein (IgM, IgG) as analyte and (iii) the specific probe as ligand and the COVID-19 virus single-standard ribonucleic acid (RNA) as analyte. After the investigation, the sensitivity of the proposed sensor was found to provide 183.33°/refractive index unit (RIU) in SPR angle (*θ_SPR_*) and 833.33THz/RIU in SPR frequency (SPRF) for detection of the COVID-19 virus spike RBD; the sensitivity obtained 153.85°/RIU in SPR angle and 726.50THz/RIU in SPRF for detection of the anti-spike protein, and finally, the sensitivity obtained 140.35°/RIU in SPR angle and 500THz/RIU in SPRF for detection of viral RNA. It was observed that whole virus spike RBD detection sensitivity is higher than that of the other two detection processes. Highly sensitive two-dimensional (2D) materials were used to achieve significant enhancement in the Goos-Hänchen (GH) shift detection sensitivity and plasmonic properties of the conventional SPR sensor. The proposed sensor successfully senses the COVID-19 virus and offers additional (1 + 0.55) × *L* times sensitivity owing to the added graphene layers. Besides, the performance of the proposed sensor was analyzed based on detection accuracy (DA), the figure of merit (FOM), signal-noise ratio (SNR), and quality factor (QF). Based on its performance analysis, it is expected that the proposed sensor may reduce lengthy procedures, false positive results, and clinical costs, compared to traditional sensors. The performance of the proposed sensor model was checked using the TMM algorithm and validated by the FDTD technique.

## 1. Introduction

The severe acute respiratory syndrome coronavirus 2 (SARS-CoV-2) is a newly reported human transferrable virus [1]. To date, more than 160 million positive cases of SARS-CoV-2 have been identified globally, causing about 3.25 million deaths [1]. The World Health Organization (WHO) declared the outbreak of the human transferrable SARS-CoV-2 virus a global pandemic [1]. Typically, the SARS-CoV-2 is a single-stranded RNA virus containing four significant proteins: (i) spike (S) glycoprotein, (ii) envelope (E), (iii) membrane (M), and (iv) nucleocapsid (N) protein [2]. The spike proteins are two types of subunit (S1, S2) protein, where S1 binding with the host cell receptor human angiotensin-converting enzyme 2 (ACE2) and S2 is liable for membrane fusion [3,4]. ACE2 is found in the human lung, kidneys, heart, and various organs, which allows the virus spike protein to enter the cell [2]. The activation of the virus spike protein causes massive damages in the brain, lung, heart, and kidneys [5]. Thus, neutralization of SARS-CoV-2 virus spike protein is a vital target in clinical research for the invention of vaccines and the development of detection procedures to reduce the spread of infectious diseases. There have been more than 58 vaccines invented to neutralize the SARS-CoV-2 virus spike receptor-binding domain (RBD) in clinical trials [6]. Some vaccines have more than 90% effectiveness against the SARS-CoV-2 virus [6,7]. The COVAX (founded by WHO, CEPI, and GAVI) is working to accelerate the development, commercial manufacture, and equitable access of SARS-CoV-2 vaccines [6]. Yet, SARS-CoV-2 viruses are progressively growing—in the UK, a new variant of the coronavirus has been identified with 17 potential changes or mutations [8]. Vital changes in an N501Y mutation in the SARS-CoV-2 virus spike protein bind with the human ACE2 receptor, and this new variant virus is a 70% more effective human transferrable virus [8]. Since the vaccine is not commercially available everywhere and with unprecedented demand for rapid detection kits to detect the SARS-CoV-2 virus, early detection and supervision are vital for controlling the pandemic. It is essential to offer tremendously sensitive, quick-test results, and low-price analytical tools to monitor affected persons for effective quarantine and timely treatment.

At present, the real-time reverse-transcriptase-polymerase chain reaction (RT-PCR) technique is used as a reference method to detect the SARS-CoV-2 virus. The RT-PCR technique takes 1–3 days to get results (generally, the test period is about 4 h) [9]. The virus ribonucleic acid (RNA) extraction is a lengthy process that affects detection accuracy. To exceed the long process of RNA extraction, Zhao et al. established a pcMNPs technique for virus RNA extraction (the test period is about ~30 min) [10]. The pcMNPs for RNA extraction is willingly announced as the subsequent RT-PCR RNA extraction [10]. The RT-PCR sensitivity ranges among 45–60% for RNA extraction; the sensitivity is increased 90–95% using pcMNPs with the specificity of identification, and enhances scientific investigation [9,10]. In the rapid test method, the COVID-19 IgG/IgM rapid test kit is used clinically to detects antibodies (IgG/IgM), where IgG is sensible after 3–6 days and IgM sensible after 8 days in affected patient blood [11,12,13]. The SARS-CoV-2 antigen rapid test kit is also applied clinically to detect the COVID-19 virus but less sensitive than the reverse-transcriptase-polymerase chain reaction (RT-PCR) technique [14,15]. Additionally, the test results are confirmed after checking the chest computed tomography (CT) scan. The rapid test kit provides false results without proper handling [16,17]. In reference [18,19,20,21], an SPR-originated fast, accurate, and highly sensitive biosensor was proposed to detect the COVID-19 virus in the future. SPR is a conventional technique applied over the last three decades to detect biomolecular interactions for clinical or research purposes [22,23]. There are numerous industrially developed SPR sensors, such as compact SPR (CSPR) biosensor, optical sensing SPR (OSSPR) biosensor, and localized SPR (LSPR) biosensor, used in biosensing applications [24]. There is no crucial difference in operating principles between the biosensors. The SPR sensor is well-known as a bio-analytical process for real-time detection of ligand and analyte binding onto the sensing region [23]. The refractive index (RI) varies because of the binding of different concentrations of the analyte [25].

In this paper, a graphene-coated five-layer SPR biosensor was proposed for the rapid detection of coronavirus. The performance of the proposed senor was analyzed numerically, ensuring early-stage rapid detection and reduction in processing time. It is expected that the proposed sensor will be tested experimentally in the near future to identify the COVID-19 virus practically. The motive of the proposed sensor is to detect the SARS-CoV-2 virus spike protein rapidly without false reports. The performance of the proposed sensor has been investigated numerically with different ligand–analytes such as antibodies–spike protein, spike protein–anti-spike protein, and probe RNA–virus single-standard RNA. The graphene implicated specific ligands can be immobilized with the target analytes. The proposed sensor can specifically detect the COVID-19 virus spike protein, antibodies against the virus spike protein, and virus single-standard RNA. Moreover, by adding a graphene film onto the sensor surface, the sensor’s sensitivity is enhanced enormously. The modified graphene coating sheet with specific ligands or gene sequences sensing transduction offers an alternative optimistic result for the experimental SARS-CoV-2 diagnosis. The main contributions of this article are as follows:

- A five-layer SPR biosensor is proposed for rapid detection of COVID-19 virus and to tackle the lengthy, expensive, false-positive results problems;
- 2D materials are employed to achieve significant enhancement in the GH shift detection sensitivity and plasmonic properties of the proposed sensor;
- A graphene layer is used to increase interaction with the ligands. The ligands can be immobilized with a graphene film through 1-pyrene butyric acid n-hydroxy-succinimide ester (PBSE) and can interact with target analytes;
- The performance of the proposed sensor is investigated with different ligand–analytes to identify the COVID-19 virus. The proposed sensor can detect the COVID-19 virus spike protein and anti-spike protein rapidly. However, the detection of virus single-standard RNA is a lengthy process because of the RNA extraction process;
- Finally, the comprehensive simulation results are analyzed during the adsorption of target analytes. The results show the detection accuracy, sensitivity, and superiority of the proposed sensor for the early-stage detection of COVID-19.

The paper is organized into five sections. The proposed sensor design model, fabrication process, FDTD method, and TMM algorithm for the proposed sensor are described in Section 2. The quality enhancement of the sensor is briefly described in Section 3. Simulation results and analysis for detection of the SARS-CoV-2 virus are represented in Section 4, and the conclusion is given in Section 5.

## 2. Methodology

**Figure 1:**
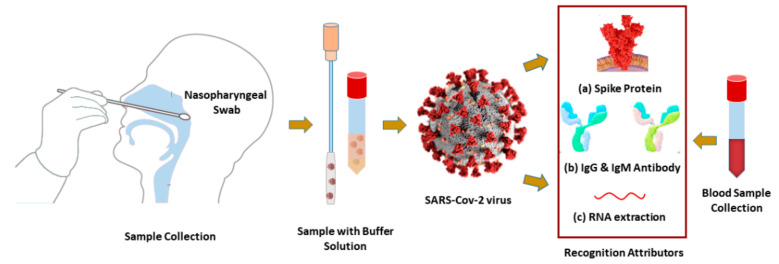
Schematic diagram of diagnosis biomarkers: (**a**) SARS-CoV-2 virus spike protein RBD, (**b**) IgG or IgM antibodies, and (**c**) or RNA-oligonucleotides are collected from nasopharyngeal swabs or human blood.

Coronavirus samples (virus spike RBD or RNA) and anti-spike protein (IgG or IgM) can be collected from nasopharyngeal swabs (NPS) and blood, respectively. They are placed immediately into viral transport media (VTM) kits, as illustrated in Figure 1.

The optimization method used in [12,26,27,28] can be used for the synthesis and purification of SARS-CoV-2 spike, and human monoclonal antibodies (IgG or IgM). The SARS-CoV-2 spike or antibodies (IgG or IgM) can flow with the phosphate-buffered saline (PBS) onto the sensor surface as an analyte [12,29]. The proposed sensor can detect the COVID-19 virus by utilizing the samples in three different modes: (i) the mAbs as ligand and the virus spike RBD as analyte, (ii) the virus spike RBD as ligand and the virus anti-spike protein as analyte and (iii) the specific probe as ligand and the virus single-standard RNA as analyte onto sensing region. In the rapid detection process of (i) and (ii), the probe ligand can be immobilized onto the graphene film through PBSE [30]. In the process of (iii), this sensor can identify the hybridization event between the probe and the virus RNA sequences. The virus RNA detection process is lengthy because of the RNA extraction process. The RI is increased due to the different concentrations of ligand–analyte binding events. The detection biomarker varies with the variation of RI and concentration. The change in reflectance (*R*), transmittance (*T*) intensity, a shift in SPR angle (*θ_SPR_*), and SPRF is calculated to recognize the presence of the COVID-19 virus.

### 2.1. The Design of the Proposed Sensor

**Figure 2:**
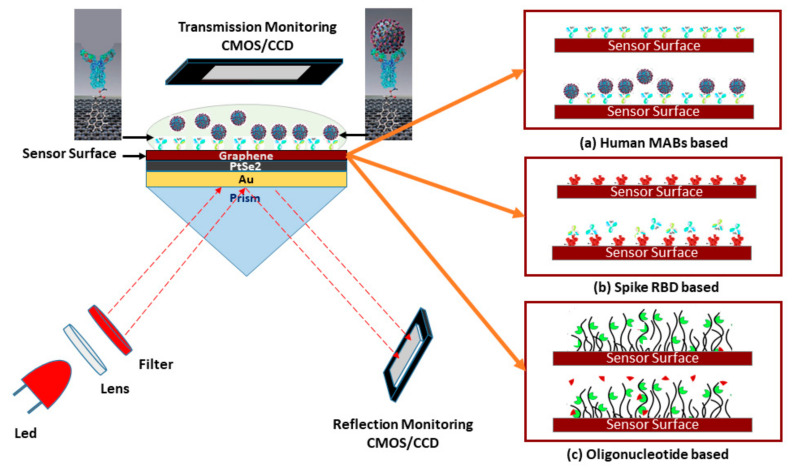
Schematic diagram of the five-layered (Bk_7_/Au/PtSe_2_/Graphene/PBS) SPR biosensor for diagnosis of SARS-CoV-2 cultured virus; three operating modes are represented to detect the SARS-CoV-2 virus: (**a**) rapid recognition of whole virus spike RBD with immobilized human mAbs (mAbs as ligand and spike RBD as analyte), (**b**) rapid recognition of mAbs with immobilized virus spike RBD (spike RBD as ligand and mAbs as analyte), and (**c**) or recognition of the virus RNA sequence with immobilized probe sequence onto the graphene implicated sensor surface.

The proposed SPR sensor for the rapid detection of the SARS-CoV-2 virus is illustrated in Figure 2. The five-layer sensing region of the sensor is based on the Kretschmann-Raether configuration. The monochromatic He–Ne laser light incident at an acceptance angle onto prism (${\mathrm{Bk}}_{7}$) and CCD (charge-coupled device) or CMOS (complementary metal oxide semiconductor) is a monitoring device. In the sensing region, the first layer is ${\mathrm{Bk}}_{7}$ and the second layer is a thin gold $\left(\mathrm{Au}\right)$ film; RI of Au is deliberated as $n_{Au}=0.1726+3.4218\;i\;$and layer thickness of $d_{Au}=50\;\mathrm{nm}$ [22,23]. Au film placed on ${\mathrm{Bk}}_{7}$ prism coupler, RI of ${\mathrm{Bk}}_{7}$ prism is $n_{BK_{7}}=\;1.5151$. The third layer is platinum-di-selenide $\left({\mathrm{PtSe}}_{2}\right)$, RI of ${\mathrm{PtSe}}_{2}$ is deliberated as $n_{PtSe2}=\;2.9189+0.9593\;i\;$and coating thickness of $d_{PtSe2}=\;2\;\mathrm{nm}$ [31]. The fourth layer is graphene, RI of the graphene layer is deliberated as $n_{g}=\;3+1.1491\;i$ and coating thickness of $d_{g}=\;0.34\times L\;\mathrm{nm}$, where L is the number of graphene layers [23]. The PBS (pH~7.4) can be used as a VTM, RI of PBS is deliberated as$\;n_{s}\;=\;1.3348+\Delta n$, where Δn is fluctuating, due to the ligand-analyte interaction on the sensing surface.

To be specific, the purpose of the BK_7_ is the highest accumulation of the incident light at the metal surface and to generate a surface plasmon wave (SPW) on the surface of the metal-dielectric interface. A gold layer and highly sensitive 2D materials (PtSe_2_ and Graphene) are employed to form plasmonic sensing substrates [32,33]. The PtSe_2_ layer is an emerging 2D group of 10 TMDC (transition metal dichalcogenides) that has intriguing optical attributes, tunable bandgap, phase transition, and superior electron mobility [34]. The graphene layer is also used for high conductivity, high carrier mobility, and superior interaction quality with the ligands. The PtSe_2_ and graphene layer synthesis enhanced the Goos-Hänchen (GH) shift detection sensitivity [34]. Therefore, the heterostructure metal interface (Au/PtSe_2_/Graphene) is used to achieve significant enhancement in detection sensitivity and plasmonic properties of the conventional SPR sensor [33,34,35].

### 2.2. The Fabrication Process of the Proposed Sensor

**Figure 3:**
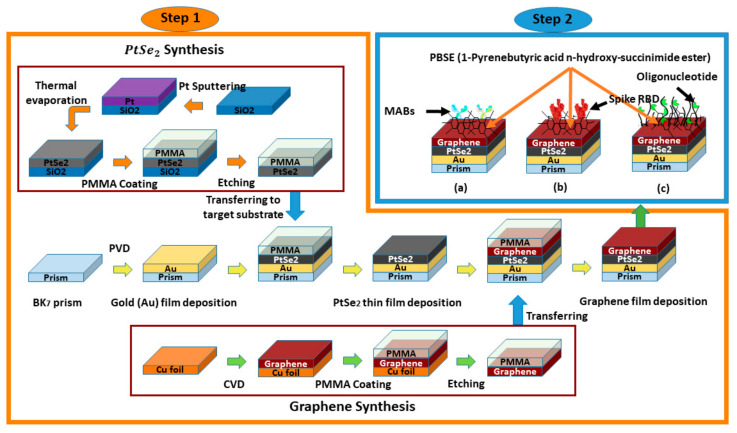
Schematic diagram of the possible multi-layer film fabrication process of the proposed SPR sensor for detection of the SARS-Cov-2 virus;—in step 1, the sensor Bk_7_/Au/PtSe_2_/graphene layers are deposited sequentially, the synthesis technique of PtSe_2_ and graphene film are also depicted. In step 2, the ligands;—(**a**) mAbs, (**b**) spike RBD, and (**c**) probe oligo are immobilized with the graphene layer through PBSE.

The possible fabrication technique of the proposed sensor for future development can be accomplished in two steps, which are illustrated in Figure 3. To demonstrate, in the first step, a piece of BK_7_ glass substrates is taken that has a x span of 7.95 μm, y span of 2 μm, and z span of 1 μm. The BK_7_ substrate is then washed in piranha solution (H_2_O_2_:3 H_2_SO_4_) to eliminate any pollutants. Then, the Au layer is grown on the top of the BK_7_ substrates using the physical vapor deposition (PVD) or sputtering technique [36,37]. The thickness of the Au layer relies on the particle sputtering deposition time [38]. The PVD method is usually renowned for representing corrosion, attire resistance, and esthetic features of the implicating films, which could be tuned on requirement [39].

The third layer can be formed of ${\mathrm{PtSe}}_{2}$ by (i) sputtering the platinum (Pt) onto the SiO_2_ substrate and (ii) SiO_2_/Pt layer can be selenized with selenium (Se) steam (at 400 °C under 150 sccm of 10% H_2_/Ar flow) by thermally assisted conversion (TAC) for two hours [40,41,42,43]. (iii) The polymethyl methacrylate (PMMA) layer can be assisted with a SiO_2_/Pt stack in a spin-coated process to transfer the ${\mathrm{PtSe}}_{2}$ film onto the Au layer. (iv) The SiO_2_ layer can be removed by a wet etching process using 2 M NaOH at 25 °C. (v) The developed PMMA/${\mathrm{PtSe}}_{2}$ stack can be cleaned in de-ionized (DI) water, transferred onto the BK_7_/Au substrate, and PMMA can be removed by applying acetone ((CH_3_)_2_CO) [40,44,45,46]. Then, the possible graphene film synthesis onto the ${\mathrm{PtSe}}_{2}$ layer includes the following steps [47,48,49,50,51]: (i) A high-quality graphene film (5 layers × 0.34 nm = 1.7 nm) can be grown onto the copper (Cu) foil by chemical vapor deposition (CVD) method at 1000 °C under a chamber pressure of 3.6 Torr by using methane (CH₄) gas as a carbon source for five minutes [47,48,49,50,51]. The CH₄ and Ar/H_2_ gas flow ratio can be maintained by operating the control valve [47]. (ii) The PMMA layer can be assisted with a Cu/graphene stack at a spin-coated process (spin rate 2000 r/min, at 175 °C) for five minutes to transfer the graphene film onto the target substrate. (iii) The Cu foil can be etched by the sulfuric acid (H_2_SO_4_) at 25 °C. (iv) The developed PMMA/graphene stack can be cleaned in DI water, transferred onto the BK_7_/Au/PtSe_2_ substrate, and PMMA can be removed by applying (CH_3_)_2_CO. In step 2, the possible immobilization process of the specific probe onto the graphene film for future development is as follows: A specific probe (sh) and the mismatching (wt) oligonucleotide solution can be taken, which has an equal concentration of 0.3 µM, and the target matching (mr) oligonucleotide solution has a concentration from 0.15 µM to 0.3 µM. The PBSE can be dissolved in (CH_3_)_2_SO (DMSO) to prepare a proper linker reagent that has a concentration of 5 mM. For possible immobilization of PBSE onto the graphene layer, the linker reagent can be added onto the graphene film for two hours at 25 °C. The immobilized graphene film by PBSE can be dissolved in probe oligonucleotide for four hours at 25 °C to confirm enough conjugation among the probe and the PBSE [52]. Similarly, the possible immobilization of antibody or spike protein onto the graphene film layer is as follows: The graphene film can be moistened in 2 mM PBSE in CH₃OH for one hour at 25 °C and cleaned many times with PBS (pH 7.4) solution with DI water. After that, the functionalized graphene film can be exposed to 250 μg/mL ligands (mAbs, or Spike RBD) for 4 h [30].

### 2.3. FDTD Technique for the Proposed Sensor

The finite-difference time-domain (FDTD) technique is used to evaluate electromagnetic field analysis for the proposed sensor by using commercial Lumerical FDTD software. FDTD technique is generally solving Maxwell’s equations using YEE-algorithms [53]. In this simulation, the non-uniform mesh is used to increase accuracy, minimizing numerical dispersion [54].

**Figure 4:**
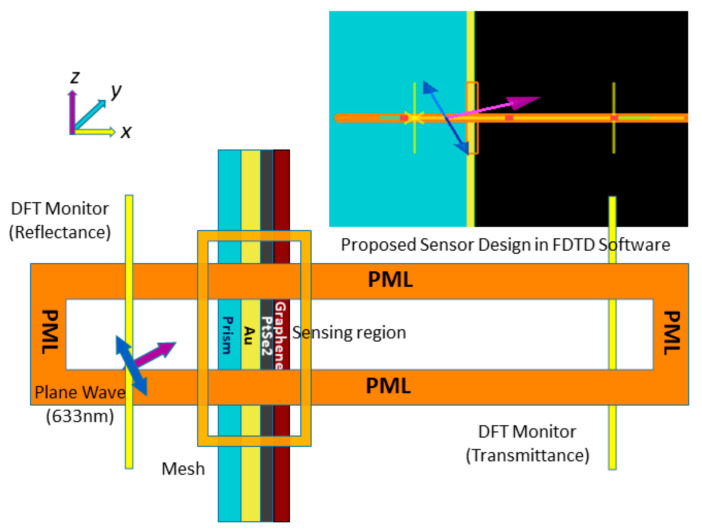
FDTD simulation schematic for the proposed SPR sensor.

The sensor surface plasmon polaritons (SPPs) excitation is evaluated by applying angular interrogation technique at a 633 nm plane wavelength [53,55]. In the FDTD simulation region, the perfectly matched layer (PML) absorbing boundary conditions are used to absorb source light with minimum reflections, and the DFT transmission monitor is used to estimate the transmittance. The simulation time setting is set to 1000 (fs) at 26.5 °C temperature, background refractive index is set to 1.00, and incident angle sweep from 30° to 70° angle. In a three-layer sensor model, BK_7_ has a geometrical position from −8 μm to −50 nm (x span of 7.95 μm), Au layer from −50 m to 0 nm (x span of 50 nm) linear to the *x*-axis. In a four-layer model, the graphene layer is added with the three-layer model; it has a geometrical location from 0 nm to 1.7 nm (span of 1.7 nm) linear to the *x*-axis. In addition, the developed five-layer model has BK_7_ with a geometrical position from −8 μm to −50 nm (x span of 7.95 μm), Au layer from −50 m to 0 nm (x span of 50 nm), PtSe_2_ layer from 0 nm to 2 nm (x span of 2 nm), and graphene layer from 2 nm to 3.7 nm (x span of 1.7 nm) linear to the *x*-axis. The developed sensor design in FDTD software is depicted in Figure 4, and the simulation result is manifested in Section 3.

### 2.4. Transfer Matrix Algorithm

According to the best performance of the proposed SPR sensor at a wavelength $\left(\lambda_{light}\right)$ of 633 nm, monochromatic laser light is applied [16,56]. The reflection intensity of p-polarized incident light can be denoted as [22,53,57,58]:

(1) $$R_{p}=\left|r_{p}^{2}\right|$$

(2) $$\begin{array}{cc} \mathrm{where}, & r_{p} \end{array}=\frac{\left(F_{11}+F_{12}n_{N}\right)n_{1\;}-\left(F_{21}+F_{22}n_{N}\right)}{\left(F_{11}+F_{12}n_{N}\right)n_{1\;}+\left(F_{21}+F_{22}n_{N}\right)}$$

The proposed sensor works on the principle of the TIR technique; however, for analyzing the multi-layer sensor numerically or to determine the reflection and transmission intensity of the proposed sensor, the TMM algorithm is encrypted in MATLAB software. For the multi-layer coated sensor, $F_{ij}$ represents the transfer matrix features as follows [22,57,58]:

(3) $$F_{ij}={\left[\overset{N-1}{\underset{K=2}{\prod }}\left(\begin{array}{cc} \cos\beta_{k} & -\frac{i\sin\beta_{k}}{n_{k}}\\ -in_{k}\sin\beta_{k} & \cos\beta_{k} \end{array}\right)\right]}_{ij}=\left[\begin{array}{cc} F_{11} & F_{12}\\ F_{21} & F_{22} \end{array}\right]$$

$$\begin{array}{cc} \mathrm{where}, & \left[\begin{array}{c} \beta_{k}=\frac{2\pi d_{k}}{\lambda }{\left(\varepsilon_{k}-n_{1}^{2}\;\sin^{2}\theta_{1}\right)}^{1/2}\\ \theta_{k}=cos^{-1}\frac{{\left(\varepsilon_{k}-n_{1}^{2}\;\sin^{2}\theta_{1}\right)}^{1/2}}{\epsilon }\\ q_{k}={\left(\frac{\mu_{k}}{\varepsilon_{k}}\right)}^{1/2} \end{array}\right. \end{array}$$

In (3), $\varepsilon_{k}$ is the dielectric constant, $\beta_{k}$ is the random phase constant, $n_{k}$ is the RI, $\theta_{k}$ is the angle of entrance, and $d_{k}$ is the depth of *k*th layer. The propagation constant ($k_{spw}$) of SPW is changed with the immobilization of analytes in the sensing region. This propagation constant can be expressed as [25,56,59]:

(4) $$k_{spw}=\frac{2\pi }{\lambda_{light}}\sqrt[{3}]{\frac{n_{Au}^{2}n_{PtSe_{2}}^{2}n_{g}^{2}}{n_{Au}^{2}+n_{PtSe_{2}}^{2}+n_{g}^{2}}}$$

The propagation constant point is shifted due to binding with the analyte as a result of the impact on surface plasmon resonance frequency (SRF). The Fresnel concept is applied in this proposed five-layered SPR sensor model to estimate transmittance intensity versus the SPRF curve. SPRF can be denoted as [22,56]:

(5) $$\mathrm{SRF}=\;\frac{C\times k_{spw}}{2\pi \;\sqrt[{3}]{n_{Au}^{2}n_{PtSe_{2}}^{2}n_{g}^{2}}}$$

The ratio of reflectance and incidence angle is familiar as the SPR curve. The incident angle is recognized as the $\theta_{SPR}$, which can be expressed as [23,60]:

(6) $$\theta_{SPR}=\sin^{-1}\left(\frac{1}{n_{BK_{7}}}\sqrt[{3}]{\frac{n_{Au}^{2}n_{PtSe_{2}}^{2}n_{g}^{2}}{n_{Au}^{2}+n_{PtSe_{2}}^{2}+n_{g}^{2}}}\right)$$

In (4)–(6), De Moivre’s method [61] is applied to calculate the *nth* roots of a complex number. In (7), $\Delta \theta_{SPR}$ and $\Delta n_{s}$ is the change of SPR angle and RI, respectively. The resonance wavelength $\left(\Delta \lambda_{res}\right)$ and the spectral resolution $\left(\Delta \lambda_{dr}\right)$ is used to calculate Δn [62]. The most commonly used mathematical formulas are as follows:

(7) $$\left[\begin{array}{c} \begin{array}{c} \Delta n=\frac{\Delta n_{s}\times \Delta \lambda_{dr}}{\Delta \lambda_{res}}\\ S=\frac{\Delta \theta_{SPR}}{\Delta n_{s}}=\;\frac{\Delta \lambda_{res}}{\Delta n_{s}} \end{array}\;\\ \begin{array}{c} \mathrm{FWHM}=\Delta \theta_{0.5}\;\\ \mathrm{DA}=\frac{1}{\Delta \theta_{0.5}} \end{array}\\ \begin{array}{c} \mathrm{FOM}=\;S\;\times DA\\ \begin{array}{c} \mathrm{SNR}=\;\frac{\Delta \theta_{SPR}}{\mathrm{FWHM}}\\ \mathrm{QF}=\mathrm{SNR}\times S \end{array} \end{array} \end{array}\right.$$

Finally, the proficiency of the SPR sensors is categorized on the basis of sensitivity (*S*), full width at half maximum (FWHM), DA, FOM, SNR, and QF parameters [31,63].

## 3. Quality Enhancement of the Proposed Sensor

**Figure 5:**
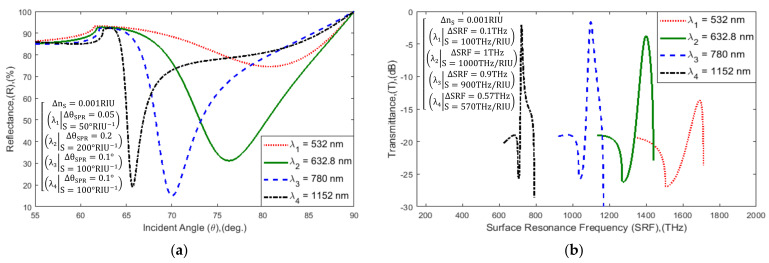
Schematic diagram of: (**a**) SPR and (**b**) SPRF curve characteristics of Bk_7_/Au(50 nm)/PtSe_2_(2 nm)/Graphene(1.7 nm)/PBS (RI = 1.3348) layered model with variation in incident light wavelength $\left(\lambda_{light}\right)$.

In the beginning, the proposed hybrid sensor performance was analyzed at different excitation wavelengths. Commercially available SPR biosensors such as Biacore-3000, SPRm-200, and Spreeta-2000 operate at a wavelength of 690 nm, 780 nm, and 830 nm, respectively [64,65]. The RI of sensor materials changes during the different excitation wavelengths, which is tabulated in Table 1 [34]. In Figure 5, the proposed sensor is operated at four different widely used wavelengths of 532 nm, 632.8 nm, 780 nm, and 1152 nm. The sensor SPR $(\theta_{SPR}$~$R_{min})$, SRF $\left(SRF\sim T_{max}\right)$ curve characteristics, and sensitivity at different operating wavelengths are tabulated in Table 2. The table highlights that the maximum sensitivity of the sensor is 200°/*RIU* and 1000*THz/RIU* at a 632.8 nm wavelength. Therefore, the proposed sensor is operated at a wavelength of 632.8 nm He-Ne laser light.

**Figure 6:**
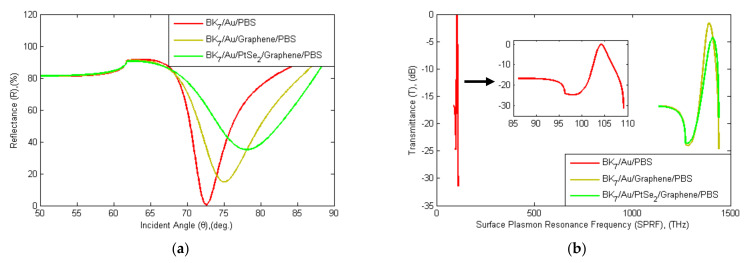
Schematic diagram of (**a**) SPR and (**b**) SPRF curve characteristics of the Bk_7_/Au(50 nm)/PtSe_2_(2 nm)/Graphene (1.7 nm) sensor with other conventional sensors with PBS analyte (RI = 1.3348).

As a second factor, the proposed SPR sensor is compared with other conventional sensors, as shown in Figure 6. In addition, Table 3 shows the evaluated results for minimum reflectance ($R_{min}$), SPR angle ($\theta_{SPR})$, maximum transmittance $\left(T_{max}\right)$, surface resonance frequency (SRF), sensitivity, FWHM, DA, FOM, and QF for three different structures of the SPR sensor model ($S^{M})$. In a three-layer model (Bk_7_/Au/PBS) without a graphene layer, $R_{min}$ is 0.54%, $\theta_{SPR}$ is 72.60°, SRF is 104.19 THz, and $T_{max}$ is 0.054 dB. Similarly, in a four-layer graphene-coated model (Bk_7_/Au/Graphene/PBS) where $R_{min}$ is 14.8%, $\theta_{SPR}$ is 75.05°, SRF is 1391.5 THz, and $T_{max}$ is 1.601 dB. Eventually, the proposed sensor will be covered with a total of five layers containing PtSe_2_ and a graphene film layer (Bk_7_/Au/PtSe_2_/Graphene/PBS), where $R_{min}$ is 35.08%, $\theta_{SPR}$ is 78.10°, SRF is 1409.3 THz and $T_{max}$ is 4.320 dB. Equation (7) is used to calculate the sensitivity, where $\Delta n_{s}\;$= 0.03 RIU. The proposed sensor’s sensitivity is enhanced to 183.3 deg RIU^−1^. The proposed five-layer $\left(S^{5}\right)$ sensor sensitivity is 6.50 times higher than that of the three-layer $\left(S^{3}\right)$ conventional sensor and the calculation as in (8).

(8) $$\left[\begin{array}{c} S^{M}=\frac{\Delta \theta_{SPR}}{\Delta n_{s}}\;\\ \;\;\;\;\;\;\;\;\;\;\;\;\;=\frac{\Delta \theta_{SPR}^{S^{3}}+\Delta \theta_{SPR}^{S^{5}}}{\Delta n_{s}}\\ \begin{array}{c} \;\;\;\;\;\;\;\;\;\;\;\;\;\;\;\;\;\;\;\;\;\;\;\;\;=\frac{\Delta \theta_{SPR}^{S^{3}}+\left(5.50\times \Delta \theta_{SPR}^{S^{3}}\right)}{\Delta n_{s}}\\ \begin{array}{c} \;\;\;\;\;\;\;\;\;\;\;\;\;\;\;\;=\frac{\left(1+5.50\right)\times \Delta \theta_{SPR}^{S^{3}}}{\Delta n_{s}}\\ \;\;\;\;\;\;\;\;\;\;\;=6.50\times S^{3} \end{array} \end{array} \end{array}\right.$$

**Figure 7:**
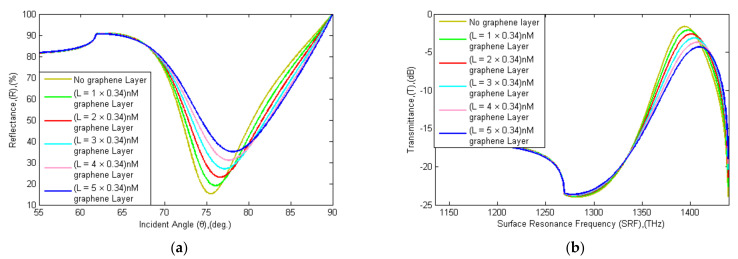
Schematic diagram of (**a**) SPR and (**b**) SPRF curve characteristics of Bk_7_/Au(50 nm)/PtSe_2_(2 nm)/Graphene (0.34 nm × *L*) coated sensor reflectance and transmittance spectra with the thickness of the graphene layers (*L* = 0, 1, …, 5) before adsorption of the analyte.

In addition, the proposed sensor’s sensitivity was increased by adding the graphene film layer (0.34 nm × *L*), where *L* is the number of additions of the graphene layer [30]. The reflectance and transmittance spectra with the addition of graphene layers is shown in Figure 7, and the data is tabulated in Table 4. The sensor’s sensitivity is enhanced by the increase in graphene layers, as previously described [69,70,71]. The sensitivity ($S^{L}$) calculation with the addition of a different number of graphene layers (*L* = 1, 2, …, 5) is shown in Equation (9). The sensitivity with one graphene layer is 18.33 deg RIU^−1^ and FOM is 48.24 RIU^−1^. The sensitivity with five graphene layers is 88.33 deg RIU^−1^ and FOM is 38.24 RIU^−1^. The proposed sensor sensitivity is enhanced (1 + 0.55) × *L* times with the addition of graphene layers. An observation in Table 4 points out that the sensitivity, FWHM, and QF are increased but FOM and DA are decreased with the addition of graphene layers. The ideal number of graphene layers was between 2 and 5 graphene layers for optimum sensitivity, QF, as well as DA and FOM [55]. In the proposed sensor, a maximum of five-layer graphene film was added to achieve maximum efficiency [31].

(9) $$\left[\begin{array}{c} S^{L}=\frac{\Delta \theta_{SPR}}{\Delta n_{s}}\;\\ \;\;\;\;\;\;\;\;\;\;\;\;\;=\frac{\Delta \theta_{SPR}^{L=0}+\Delta \theta_{SPR}^{L=1}}{\Delta n_{s}}\\ \begin{array}{c} \;\;\;\;\;\;\;\;\;\;\;\;\;\;\;\;\;\;\;\;\;\;\;\;\;=\frac{\Delta \theta_{SPR}^{L=0}+\left(0.55\times \Delta \theta_{SPR}^{L=0}\right)}{\Delta n_{s}}\\ \begin{array}{c} \;\;\;\;\;\;\;\;\;\;\;\;\;\;\;\;\;=\frac{\left(1+0.55\right)\times \Delta \theta_{SPR}^{L=0}}{\Delta n_{s}}\\ \;\;\;\;\;\;\;\;\;\;\;\;\;\;\;\;\;\;\;\;\;\;\;=\left(1+0.55\right)\times S^{0} \end{array} \end{array} \end{array}\right.$$

**Figure 8:**
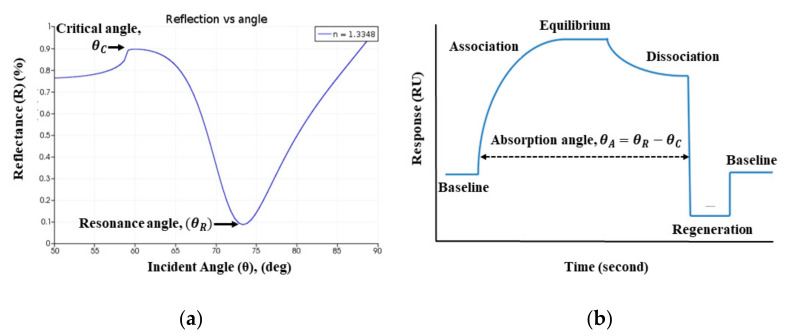
The Schematic of: (**a**) SPR, and (**b**) SPR sensorgram absorption curve.

Further, the precise relevance of the SPR curve is to the SPR sensorgram, which has been utilized clinically over three decades to identify the presence of the target analyte. The real-time absorption curve $\left(\theta_{A}\right)$ in the sensorgram is obtained from the difference of SPR resonance angle $\left(\theta_{R}\right)$ and critical angle $\left(\theta_{C}\right)$, as shown in Figure 8 [72,73]. The sensorgram curve indicates the change of RI in the sensing region due to the interaction of the target analyte [74]. In this article, SPR curve characteristics are used to identify persons infected or uninfected by the COVID-19 virus.

**Figure 9:**
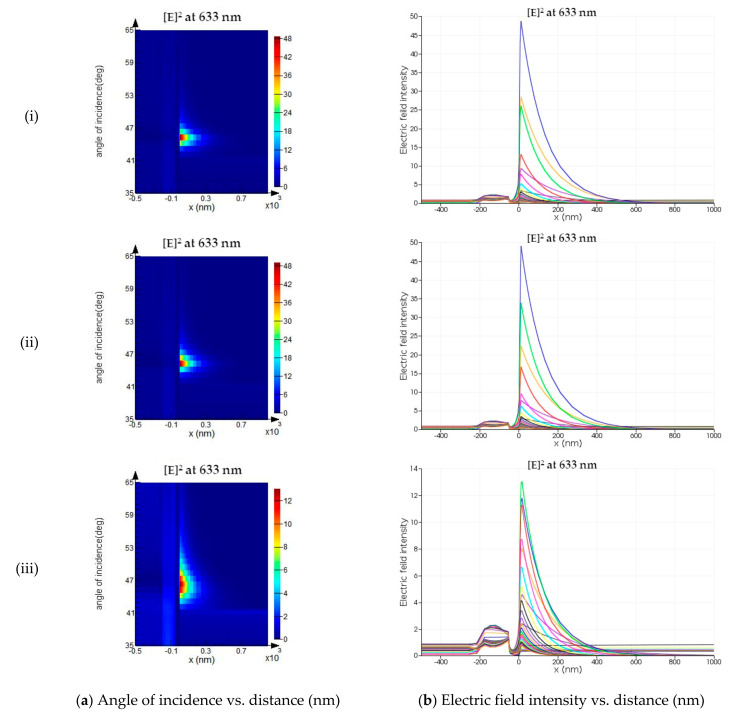
The schematic of (**a**) the angle of incidence, and (**b**) the electric field intensity as a function of normal distance from the interface for (**i**) three-layer, (**ii**) four-layer, and (**iii**) five-layer SPR sensor configuration.

Furthermore, electric field intensity is evaluated for the developed sensor, comparing it with other conventional sensors. In Lumerical FDTD, the angular interrogation technique is utilized to estimate the SPPs excitation near the sensor surface. The SPPs’ excitation produces an evanescent wave that decays exponentially with distance in a direction normal to the boundary [75]. The SPPs’ electromagnetic field intensity is maximum at a resonance angle of the incident light [75]. In Figure 9, the electric field intensity and angle of the incident concerning normal distance from the sensor interface are monitored for the three-layer, four-layer, and proposed five-layer SPR sensor. The strength of electric field intensity is enhanced near the surface in the proposed SPR sensor, compared to the other conventional sensor. Thus, the sensitivity of the proposed sensor is increased enormously for the detection of target analytes.

## 4. Result and Analysis

### 4.1. Rapid Detection of SARS-CoV-2 Virus Spike RBD

In rapid detection of the SARS-CoV-2 spike RBD, mAbs (IgG or IgM) can be immobilized onto the graphene film through PBSE as a probe ligand, and the whole virus can flow with PBS solution as an analyte. The RI of the sensing region is changed due to the interaction of different concentrations of the ligand-analyte. However, in numerical analysis, the sensing region RI is considered as, $n_{s}=1.3348+\Delta n$, where the RI of PBS (pH 7.4) is 1.3348 and Δn is fluctuating due to the different concentrations of ligand-analyte interaction onto the sensing surface. The adaptive interaction distribution algorithm (AIDA) algorithm has been used to solve the Δn following reference [76,77,78]. To realize the interaction mechanism of the SARS-CoV-2 spike RBD, a rate constant distribution (RCD) algorithm with AIDA was established in reference [76,77]. The real experimental interaction mechanism of SARS-CoV-2 spike RBD with human ACE2 is the same or almost similar to the algorithm [76]. The affinity constant (K_D_), association (k_a_), and dissociation (k_d_) rate constants are varied due to variation in ligands and analytes [78,79]. Therefore, the rate constants value at the different concentration levels (concentration of 1.95 nM to 62.5 nM) of virus spike RBD is followed, as described in reference [76,77,78].

**Figure 10:**
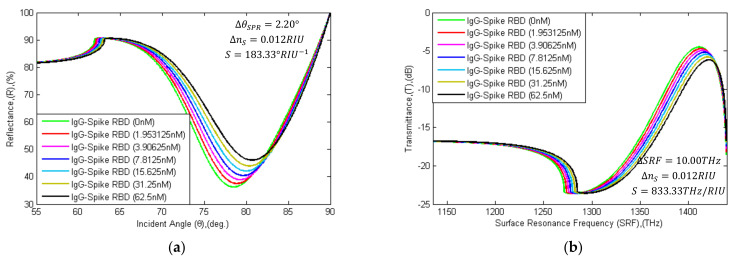
Schematic diagram of: (**a**) SPR and (**b**) SPRF characteristics of Bk_7_/Au(50 nm)/PtSe_2_(2 nm)/Graphene(1.7 nm) substrates with immobilized IgG (H014 or S309) as ligand and different concentration levels of SARS-CoV-2 spike RBDs (concentration of 1.953125 nM to 62.5 nM) as the analyte.

In Figure 10, the SPR and SPRF curve characteristics are illustrated with the different concentration levels of SARS-CoV-2 Spike RBDs flows as analyte. The $\theta_{SPR}$~$R_{min}$ and $SRF\sim T_{max}$ characteristics are calculated in Table 5, with the variation of the analyte. In the simulation, the RI of PBS solution (concentration of 500 nM) without virus spike RBD was computed using the AIDA that has flowed onto the sensor surface, where $R_{min}$ is 36.25%, $\theta_{SPR}$ is 78.50°, SRF is 1411.3 THz and $T_{max}$ is 4.5017 dB calculated, respectively. Similarly, the RI of PBS solution with SARS-CoV-2 virus spike RBD (concentration of 1.95 to 62.5 nM) that flowed onto the sensor surface was computed. The RI is changed due to the interaction of different concentrations of mAbs and SARS-CoV-2 spike RBD. Due to the absorption of 1.953125nM SARS-CoV-2 spike RBD, the detection attributors shift right by 0.35° (from 78.50° to 78.85°) and 1.8 THz (from 1411.3 THz to 1413.1 THz). Furthermore, reflectance (R) and transmittance (T) also changed by 1.27% (from 36.25% to 37.52%) and 0.2018 dB (from 4.5017 dB to 4.7035 dB). Similarly, the detection attributors change with the absorption of SARS-CoV-2 spike RBDs; otherwise, the status would remain the same.

The following (10) is employed for the diagnosis of COVID-19 patients:

(10) $$\left[\begin{array}{c} \Delta R_{min}^{i}\;\left[\%\right]=\;\left|R_{min}^{0\;\mathrm{nM}}-R_{min}^{1.953125\;\mathrm{nM}}\right|=1.27\%\\ \Delta \theta_{SPR}^{i}\;\left[\deg.\right]=\;\left|\theta_{SPR}^{0\;\mathrm{nM}}-\theta_{SPR}^{1.953125\;\mathrm{nM}}\right|=0.35\;\deg\\ \begin{array}{c} \Delta T_{max}^{i}\;\left[\mathrm{dB}\right]=\;\left|T_{max}^{0\;\mathrm{nM}}-T_{max}^{1.953125\;\mathrm{nM}}\right|=0.2018\;\mathrm{dB}\\ \Delta SRF_{freq}^{i}\;\left[\mathrm{THz}\right]=\;\left|SRF_{freq}^{0\;\mathrm{nM}}-SRF_{freq}^{1.953125\;\mathrm{nM}}\right|=1.8\;\mathrm{THz} \end{array} \end{array}\right.$$

### 4.2. Rapid Detection of SARS-CoV-2 Anti-Spike Protein

**Figure 11:**
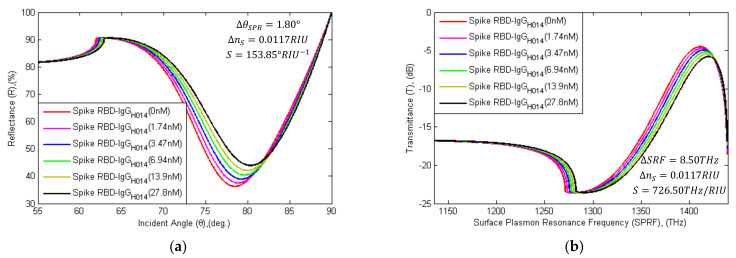
Schematic diagram of (**a**) SPR and (**b**) SPRF characteristics of Bk_7_/Au(50 nm)/PtSe_2_(2 nm)/Graphene(1.7 nm) substrates with immobilized SARS-CoV-2 spike RBDs as the ligand and different concentration levels of IgG-H014 (concentration of 1.74 nM to 27.8 nM) as the analyte.

In this pandemic, numerous SARS-CoV-2 anti-spike proteins or mAbs have been screened out with time. mAbs was found to interact with virus spike RBD at the nM level and successfully deactivate the SARS-CoV-2 virus Spike RBD. The mAbs (3F11, BD-368-2, ab1, CB6, B38, H4, P2C-1F11, rRBD-15, 311mab–31B5, 311mab–32D4, CC12.1, COVA1-18, COVA2-15, S309, ADI-55689, REGN10989, H014, COV2-2196, COV2-2130) block the interaction between virus spike RBD and ACE2 [80]. For rapid detection, SARS-CoV-2 spike RBDs can be immobilized onto the graphene film with PBSE as a probe, and the mAbs (IgG;-H014) can flow with PBS as an analyte [81]. In Figure 11, the SPR and SPRF curve characteristics are shown with the different concentration levels of H014 flows as the analyte. The $\theta_{SPR}$~$R_{min}$ and $SRF\sim T_{max}$ characteristics are calculated in Table 6.

In the simulation, the RI of PBS solution (concentration of 500 nM) without IgG (H014) was computed using the AIDA that had flowed onto the sensor surface, where $R_{min}$ is 36.25%, $\theta_{SPR}$ is 78.50°, SRF is 1411.3 THz, and $T_{max}$ is 4.5017 dB calculated, respectively. Similarly, the RI of the PBS solution with H014 (concentration of 1.74 to 27.8 nM, ref. [81]) that has flowed onto the sensor surface was computed. Due to the absorption of 1.74 nM H014, the detection attributors shift right by 0.35° (from 78.50° to 78.85°) and 1.8 THz (from 1411.3 THz to 1413.1 THz). While absorption increases, $R_{min}^{C_{IgG}}$ and $T_{max}^{C_{IgG}}$ attributors are also changed by 1.27% (from 36.25% to 37.52%) and 0.2017 dB (from 4.5017 dB to 4.7034 dB). Similarly, the detection parameters change with the absorption of H014; otherwise, the status would remain the same. The following (11) is employed for the diagnosis of COVID-19 patients:

(11) $$\left[\begin{array}{c} \Delta R_{min}^{ii}\;\left[\%\right]=\;\left|R_{min}^{0\;\mathrm{nM}}-R_{min}^{1.74\;\mathrm{nM}}\right|=1.27\%\\ \Delta \theta_{SPR}^{ii}\;\left[\deg.\right]=\;\left|\theta_{SPR}^{0\;\mathrm{nM}}-\theta_{SPR}^{1.74\;\mathrm{nM}}\right|=0.35\;\deg\\ \begin{array}{c} \Delta T_{max}^{ii}\;\left[\mathrm{dB}\right]=\;\left|T_{max}^{0\;\mathrm{nM}}-T_{max}^{1.74\;\mathrm{nM}}\right|=0.2017\;\mathrm{dB}\\ \Delta SRF_{freq}^{ii}\;\left[\mathrm{THz}\right]=\;\left|SRF_{freq}^{0\;\mathrm{nM}}-SRF_{freq}^{1.74\;\mathrm{nM}}\right|=1.8\;\mathrm{THz} \end{array} \end{array}\right.$$

### 4.3. Real-time Detection of SARS-CoV-2 RNA Sequence

**Figure 12:**
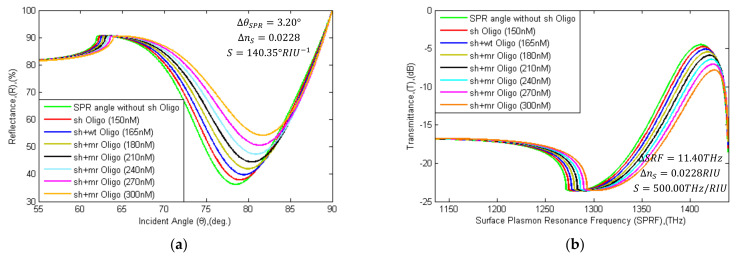
Schematic diagram of (**a**) SPR and (**b**) SPRF curve characteristics of Bk_7_/Au(50 nm)/PtSe_2_(2 nm)/Graphene (0.34 nm × L) substrates without probe (sh-Oligo), with sh-Oligo and different concentrated levels of oligonucleotide (wt or mr type) binding for recognition of SARS-Cov-2 virus RNA. $\theta_{SPR}$ angle and SPRF shift right due to binding with oligonucleotides.

Currently, the RT-PCR method is being applied to detect the SARS-CoV-2 virus. However, RNA extraction of the SARS-CoV-2 Spike is a long-time process. It takes more than one to three days to get a clinical report. To overcome this time-consuming procedure of RNA extraction, Zhao et al. developed a pcMNPs technique for RNA extraction (required ~30 min) [10]; it has been identified as a subsequent RT-PCR method. The sensor graphene layer can be attached to specific probe RNA sequences via PBSE to diagnose specific RNA sequences. This proposed sensor can detect hybridization events among the target SARS-CoV-2 spike RNA sequence and the probe RNA sequence. In Table 7, forward-reverse primers and probe genome sequences are specified with nucleotide position for detection of the RNA sequence of the COVID-19 virus [82,83,84]. In addition, the entirely matching (mr) sequence and mismatching (wt) sequence with probe (sh) linker is tabulated. In Figure 12, the SPR angle and SRF shifted right with the variation of different concentrated target RNA sequences. The detection attributor $\theta_{SPR}$~$R_{min}$ and $SRF\sim T_{max}$ are calculated with probe (sh) sequence, where $R_{min}$ is 37.92%, $\theta_{SPR}$ is 78.95°, SRF is 1413.7 THz, and $T_{max}$ is 4.7670 dB. The attributors’ shifting parameters are tabulated in Table 8.

In the simulation, the mismatching type (wt) RNA oligonucleotide was seen to flow with 500 nM PBS and binding with sh-sequence, where $R_{min}$ is 39.82%, $\theta_{SPR}$ is 78.45°, SRF is 1416.0 THz, and $T_{max}$ is 5.0778 dB calculated, respectively. The attributors are shifted in minor levels during immobilization with a wt-type sequence than immobilization with an mr-type sequence. The entirely matching type (mr) RNA sequence immobilized with sh-sequence detection attributors are shifted right by 1.0° (from 78.95° to 79.95°) and 4.4 THz (from 1413.7 THz to 1418.1 THz). Meanwhile, R and T are also increased by 4.05% (from 37.92% to 41.97%) and 0.6759 dB (from 4.7670 dB to 5.4429 dB). In the sensing region, RI is increased with the increasing concentration of mr-type sequences. If a hybridization event takes place between the probe sequences and the mr-sequences, the attributors’ shift is equal to or greater than $\Delta R_{min}^{c}$, $\Delta \theta_{SPR}^{c}$, $\Delta T_{max}^{c}$, and $\Delta SRF_{freq}^{c}$; if the event does not take place, the shift is smaller. For the detection of the virus, (12) is used:

(12) $$\left[\begin{array}{c} \Delta R_{min}^{iii}\;\left[\%\right]=\;\left|R_{min}^{150\;\mathrm{nM}}-R_{min}^{180\;\mathrm{nM}}\right|=4.05\%\\ \Delta \theta_{SPR}^{iii}\;\left[\deg.\right]=\;\left|\theta_{SPR}^{150\;\mathrm{nM}}-\theta_{SPR}^{180\;\mathrm{nM}}\right|=1.00\;\deg\\ \begin{array}{c} \Delta T_{max}^{iii}\;\left[\mathrm{dB}\right]=\;\left|T_{max}^{150\;\mathrm{nM}}-T_{max}^{180\;\mathrm{nM}}\right|=0.6759\;\mathrm{dB}\\ \Delta SRF_{freq}^{iii}\;\left[\mathrm{THz}\right]=\left|SRF_{freq}^{150\;\mathrm{nM}}-SRF_{freq}^{180\;\mathrm{nM}}\right|=4.4\;\mathrm{THz} \end{array} \end{array}\right.$$

In the numerical study, the SARS-CoV-2 virus spike RBD detection method is suggested by using the proposed sensor because monoclonal antibodies (IgG, IgM) take 3–8 days to be produced in the human body [85,86] and the RNA extraction period is a time-consuming process [10]. The detection of whole SARS-CoV-2 spike RBD and the anti-spike protein is done by a rapid method, compared to the virus RNA detection technique. In addition, the proposed sensor sensitivity provides 183.33° RIU^−1^ and 833.33 THz RIU^−1^ for detection of the whole SARS-Cov-2 virus spike; the sensor sensitivity offers 153.85° RIU^−1^ and 726.50 THz RIU^−1^ for detection of antibodies (IgG or IgM) against the virus spike protein, and the sensor sensitivity obtains 140.35° RIU^−1^ and 500.0THz RIU^−1^ for detection of virus RNA. The final decision or tested result will depend on the conditions of the attributor$(\theta_{SPR}$~$R_{min}$ and $SRF\sim T_{max}).$ Eventually, the patient’s COVID-19 clinical test result is contingent on detection of attributors characteristics (Table 9). In rapid recognition of the whole SARS-CoV-2 virus spike RBD procedure, if $\Delta R_{min}^{i}\sim \Delta \theta_{SPR}^{i}$ or $\Delta T_{max}^{i}\sim \Delta SRF_{freq}^{i}$ is larger or equivalent to $\Delta R_{min}^{SC2R}\sim \Delta \theta_{SPR}^{SC2R}$ or $\Delta T_{max}^{SC2R}\sim \Delta SRF_{freq}^{Sc2R}$ then the COVID-19 result is positive. Similarly, if $\Delta R_{min}^{i}\sim \Delta \theta_{SPR}^{i}$ or $\Delta T_{max}^{i}\sim \Delta SRF_{freq}^{i}$ is smaller than $\Delta R_{min}^{SC2R}\sim \Delta \theta_{SPR}^{SC2R}$ or $\Delta T_{max}^{SC2R}\sim \Delta SRF_{freq}^{Sc2R}$, then the COVID-19 result is negative. The detection of antibodies and RNA hybridization events against the SARS-CoV-2 virus follow the same procedure. In Table 9, the *X* and *Y* parameters are identified by the detection method. This sensor shows robust performance for the rapid detection of the SARS-CoV-2 virus.

## 5. Conclusions

This paper proposes a graphene-based multi-layer coated SPR sensor for the early-stage detection of the COVID-19 virus. The proposed sensor was analyzed for rapid diagnosis and effectively differentiates between infected or uninfected persons. The BK_7_/Au/PtSe_2_/Graphene film-coated SPR sensor was found to have a superior sensitivity of 183.3°/RIU, compared to other conventional sensors. Graphene films have high conductivity and interaction quality with the ligand. The sensor sensitivity was found to be enhanced (1 + 0.55) × *L* times by increasing the number of graphene layers. The proposed sensor utilized the virus spike RBD, antibodies (IgG or IgM), or virus RNA to detect the virus. The performance of the proposed sensor was confirmed by the TMM algorithm and validated by the FDTD technique. Numerically, the proposed senor ensures early-stage detection, reducing processing time without false results. The proposed sensor is expected to be implemented commercially or clinically to identify COVID-19 patients.

## Figures and Tables

**Table 1:** RI of sensor materials at different wavelengths.

RI	$\lambda_{1}\;(532.0\;\mathrm{nm})$	$\lambda_{2}\;(632.8\;\mathrm{nm})$	$\lambda_{3}\;(780.0\;\mathrm{nm})$	$\lambda_{4}\;(1152\;\mathrm{nm})$
BK_7_ [34]	1.5195	1.5151	1.5112	1.5055
Au [34,66]	0.0990 + 2.9952i	0.1377 + 3.6183i	0.2063 + 4.5133i	0.4417 + 6.7308i
PtSe_2_ [34,67]	2.5917 + 1.1332i	2.9029 + 0.8905i	2.8229 + 0.5277i	2.7986 + 0.2878i
Graphene [34,68]	3 + 0.9658i	3 + 1.1487i	3 + 1.4160i	3 + 2.0913i

**Table 2:** Data evaluation of $\Delta R_{min}^{\lambda },\;\Delta \theta_{SPR}^{\lambda },\;\Delta T_{max}^{\lambda }\;and\;\Delta SRF_{Freq}^{\lambda }$, and sensitivity (S) attributors with change of incident wavelength $\left(\lambda_{light}\right)$ in the proposed SPR sensor.

Incident Light Wavelength, (*λ*) [nm]	$R_{min}^{\lambda }\;[\%]$	$\theta_{SPR}^{\lambda }\;[\deg.]$	$T_{max}^{\lambda }\;[\mathrm{dB}]$	$SRF_{freq}^{\lambda }[\mathrm{THz}]$	$\Delta R_{min}^{\lambda }$ $[\%]=\left|R_{min}^{532\;nm}-R_{min}^{\lambda }\right|$	$\Delta \theta_{SPR}^{\lambda }$ $[\deg.]=\left|\theta_{SPR}^{532\;nm}-\theta_{SPR}^{\lambda }\right|$	$\Delta T_{max}^{\lambda }$ $[\mathrm{dB}]=\left|T_{max}^{532\;nm}-T_{max}^{\lambda }\right|$	$\Delta SRF_{Freq}^{\lambda }$ $[\mathrm{THz}]=\left|SRF_{Freq}^{532\;nm}-SRF_{Freq}^{\lambda }\right|$	Sensitivity (S)	
									$\left[\deg/\mathrm{RIU}\right]$	$\left[\mathrm{THz}/\mathrm{RIU}\right]$
$\lambda_{1}=\;$532.0 nm	74.56	80.70	13.689	1691.2	0.00	0.00	0.0000	0.00	50	100
$\lambda_{2}=\;$632.8 nm	31.22	76.25	3.7420	1399.6	43.34	4.45	9.9470	291.60	200	1000
$\lambda_{3}=\;$780.0 nm	15.04	70.05	1.6288	1098.6	59.52	10.65	12.0602	592.60	100	900
$\lambda_{4}=\;$1152 nm	19.12	65.70	2.1217	721.29	55.44	15.00	11.5673	969.91	100	570

**Table 3:** Evaluation of the proposed sensor model with other conventional models with regards to sensitivity (S), FWHM, detection accuracy (DA), the figure of merit (FOM), and quality factor (QF).

$\mathrm{Structure}\;(S^{M})$	$R_{min}^{S}$ $\left[\%\right]$	$\theta_{SPR}^{S}$ $\left[\deg\right]$	$T_{max}^{S}$ $\left[\mathrm{dB}\right]$	$SRF_{freq}^{S}$ $\left[\mathrm{THz}\right]$	$\Delta R_{min}^{S}$ $\left[\%\right]$	$\Delta \theta_{SPR}^{S}$ $\left[\deg\right]$	$\Delta T_{max}^{S}$ $\left[\mathrm{dB}\right]$	$\Delta SRF_{freq}^{S}$ $\left[\mathrm{THz}\right]$	S $\left[\deg/\mathrm{RIU}\right]$	FW-HM $\left[\deg\right]$	DA $\left[1/\deg\right]$	FOM $\left[{\mathrm{RIU}}^{-1}\right]$	QF $\left[\deg/\mathrm{RIU}\right]$
Bk_7_/Au(50 nm)/PBS	0.54	72.60	0.054	104.19	-	-	-	-	-	-	-	-	-
Bk_7_/Au(50 nm)/Graphene (5 layer)/PBS	14.80	75.05	1.601	1391.5	14.26	2.46	1.547	1287.31	82.0	1.30	0.77	63.07	155.17
Bk_7_/Au(50 nm)/PtSe_2_ (2 nm)/Graphene (5 layer)/PBS	35.08	78.10	4.320	1409.3	34.54	5.50	4.266	1305.11	183.3	3.90	0.26	47.00	258.50

**Table 4:** Comparison of change in $R_{min}^{L},\;\;\theta_{SPR}^{L},\;T_{max}^{L},\;\;SRF_{freq}^{L}$, sensitivity, FWHM, DA, FOM, and QF with the variation of the graphene layer (L) in the proposed SPR biosensor.

Graphene layer thickness	$R_{min}^{L}\;\left[\%\right]$	$\theta_{SPR}^{L}\;$ $\left[\deg\right]$	$T_{max}^{L}\;$ $\left[\mathrm{dB}\right]$	$SRF_{freq}^{L}$ $\left[\mathrm{THz}\right]$	$\Delta R_{min}^{L}\;[\%]$	$\Delta \theta_{SPR}^{L}$ $\left[\deg\right]$	$\Delta T_{max}^{L}$ $\left[\mathrm{dB}\right]$	$\Delta SRF_{Freq}^{L}$ $\left[\mathrm{THz}\right]$	S $\left[\deg/\mathrm{RIU}\right]$	FW-HM $\left[\deg\right]$	DA [1/deg]	FOM $\left[{\mathrm{RIU}}^{-1}\right]$	QF $\left[\deg/\mathrm{RIU}\right]$
*L* = 0 × 0.34 nm	15.20	75.45	1.6409	1394.2	-	-	-	-	-	-	-	-	-
*L* = 1 × 0.34 nm	19.05	76.00	2.1133	1397.6	3.85	0.55	0.4724	3.40	18.33	0.38	2.63	48.24	26.53
*L* = 2 × 0.34 nm	22.99	76.55	2.6124	1400.9	7.79	1.10	0.9715	6.70	36.67	0.78	1.28	47.01	51.72
*L* = 3 × 0.34 nm	27.00	77.10	3.1464	1404.0	11.8	1.65	1.5055	9.80	55.00	1.22	0.82	45.08	74.39
*L* = 4 × 0.34 nm	31.04	77.60	3.7156	1406.8	15.84	2.15	2.0747	12.60	71.67	1.73	0.58	41.43	89.07
*L* = 5 × 0.34 nm	35.08	78.10	4.3201	1409.3	19.88	2.65	2.6792	15.10	88.33	2.31	0.43	38.24	101.3

**Table 5:** $\Delta R_{min}^{C_{RBD}}$$,\theta_{SPR}^{C_{RBD}},\;T_{max}^{C_{RBD}}$ and $SRF_{freq}^{C_{RBD}}$ characteristics for different concentrations of SARS-Cov-2 Spike RBD as an analyte.

$\mathrm{SARS}-\mathrm{Cov}-2\;\mathrm{RBD}\;\mathrm{Concentration}\;(C_{RBD})\;\mathrm{with}\;\mathrm{PBS}\;(500\;\mathrm{nM})\;[\mathrm{nM}]$		$R_{min}^{C_{RBD}}\;[\%]$	$\theta_{SPR}^{C_{RBD}}\;[\deg.]$	$T_{max}^{C_{RBD}}\;[\mathrm{dB}]$	$SRF_{freq}^{C_{RBD}}[\mathrm{THz}]$	$\Delta R_{min}^{SC2R}$ $[\%]=\;\left|R_{min}^{0\;nM}-R_{min}^{C_{RBD}}\right|$	$\Delta \theta_{SPR}^{SC2R}$ $[\deg.]=\;\left|\theta_{SPR}^{0\;nM}-\theta_{SPR}^{C_{RBD}}\right|$	$\Delta T_{max}^{SC2R}$ $[\mathrm{dB}]\;=\;\left|T_{max}^{0\;nM}-T_{max}^{C_{RBD}}\right|$	$\Delta SRF_{freq}^{SC2R}$ $[\mathrm{THz}]=\;\left|SRF_{freq}^{0\;nM}-SRF_{Freq}^{C_{RBD}}\right|$
	0 nM	36.25	78.50	4.5017	1411.3	0.00	0.00	0.0000	0.00
	1.953125 nM	37.52	78.85	4.7035	1413.1	1.27	0.35	0.2018	1.80
	3.90625 nM	38.91	79.25	4.9280	1414.9	2.66	0.75	0.4263	3.60
RBD	7.8125 nM	40.42	79.60	5.1792	1416.6	4.17	1.10	0.6775	5.30
	15.625 nM	42.09	79.95	5.4627	1418.2	5.84	1.45	0.9610	6.90
	31.25 nM	43.94	80.30	5.7880	1419.8	7.69	1.80	1.2863	8.50
	62.5 nM	46.06	80.70	6.1728	1421.3	9.81	2.20	1.6711	10.00

**Table 6:** $\Delta R_{min}^{C_{RBD}}$$,\theta_{SPR}^{C_{RBD}},\;T_{max}^{C_{RBD}}$ and $SRF_{freq}^{C_{RBD}}$ characteristics for different concentrations of IgG (H014) as an analyte.

$\mathrm{IgG}\;\mathrm{Concentration}\;(C_{IgG})\;\mathrm{with}\;\mathrm{PBS}\;(500\;\mathrm{nM})\;[\mathrm{nM}]$		$R_{min}^{C_{IgG}}\;[\%]$	$\theta_{SPR}^{C_{IgG}}$ [deg.]	$T_{max}^{C_{IgG}}$ [dB]	$SRF_{freq}^{C_{IgG}}\;[\mathrm{THz}]$	$\Delta R_{min}^{IgG}[\%]\;=$ $\left|R_{min}^{0\;nM}-R_{min}^{C_{IgG}}\right|$	$\Delta \theta_{SPR}^{IgG}$ $[\deg.]=\;\left|\theta_{SPR}^{0\;nM}-\theta_{SPR}^{C_{igG}}\right|$	$\Delta T_{max}^{IgG}[\mathrm{dB}]=$ $\left|T_{max}^{0\;nM}-T_{max}^{C_{IgG}}\right|$	$\Delta SRF_{freq}^{IgG}$ $[\mathrm{THz}]=\;\left|SRF_{freq}^{0\;nM}-SRF_{freq}^{C_{IgG}}\right|$
H014	0 nM	36.25	78.50	4.5017	1411.3	0.00	0.00	0.0000	0.00
	1.74 nM	37.52	78.85	4.7034	1413.1	1.27	0.35	0.2017	1.80
	3.47 nM	38.91	79.25	4.9277	1414.9	2.66	0.75	0.4260	3.60
	6.94 nM	40.42	79.60	5.1784	1416.6	4.17	1.10	0.6767	5.30
	13.9 nM	42.08	79.95	5.4608	1418.2	5.83	1.45	0.9591	6.90
	27.8 nM	43.92	80.30	5.7837	1419.8	7.67	1.80	1.2820	8.50

**Table 7:** Primers and probe sequence orientation for recognition of SARS-CoV-2 virus RNA.

Name	Target Genes	Sequence 5′→3′	Nucleotide position	Target Genes	Sequence 5′→3′	Nucleotide Position
Forward Primer:	ORF 1ab	CCCTGTGGGTTTTACACTTAA	13342-13362	N gene	GGGGAACTTCTCCTGCTAGAAT	28881-28902
Reverse Primer:		ACGATTGTGCATCAGCTGA	13442-13460		CAG ACATTTTGCTCTCAAGCTG	28958-28979
Immobilized Mutant Probe (sh):		FAM-CCGT**CTGCGGTATGTGGAAAGGTT**ATGG-BHQ	13377-13404		FAM-TT**GCTGCTGCTTGACAGA**TT-TAMRA	28934-28953
Matching type (mr) Sequence:		TCCAA**CCTTTCCACATACCGCAG**CGGA	-		AAC**TCTGTCAAGCAGCAGC**AA	-
Mismatching type (wt) Sequence:		TCC**AAGCAAACCCAATACCGCAG**CGGA	-		AAC**TCTACTAATTAGCAGC**AA	-

**Table 8:** $R_{min}^{C_{N}},\theta_{SPR}^{C_{N}},T_{max}^{C_{N}}\mathrm{and}{\mathrm{SRF}}_{freq}^{C_{N}}$ for different concentrations of the target oligonucleotides as an analyte.

$\mathrm{Concentration}\;(C_{N})\;\mathrm{with}\;\mathrm{PBS}\;(500\;\mathrm{nM})\;[\mathrm{nM}]$	$R_{min}^{C_{N}}\;[\%]$	$\theta_{SPR}^{C_{N}}\;[\deg.]$	$T_{max}^{C_{N}}\;[\mathrm{dB}]$	$SRF_{freq}^{C_{N}}[\mathrm{THz}]$	$\Delta R_{min}^{N}$ $[\%]=\left|R_{min}^{sh}-R_{min}^{C_{N}}\right|$	$\Delta \theta_{SPR}^{N}$ $[\deg.]=\left|\theta_{SPR}^{sh}-\theta_{SPR}^{C_{N}}\right|$	$\Delta T_{max}^{N}[\mathrm{dB}]=$ $\left|T_{max}^{sh}-T_{max}^{C_{N}}\right|$	$\Delta SRF_{Freq}^{N}$ $[\mathrm{THz}]=\left|SRF_{freq}^{sh}-SRF_{freq}^{C_{N}}\right|$
0 nM (no-sh-Oligo)	36.25	78.50	4.5017	1411.3	-	-	-	-
150 nM (sh-Oligo)	37.92	78.95	4.7670	1413.7	0.00	0.00	0.0000	0.00
165 nM (wt-Oligo)	39.82	79.45	5.0778	1416.0	1.90	0.50	0.3108	2.30
180 nM (mr-Oligo)	41.97	79.95	5.4429	1418.1	4.05	1.00	0.6759	4.40
210 nM (mr-Oligo)	44.47	80.40	5.8827	1420.2	6.55	1.45	1.1157	6.50
240 nM (mr-Oligo)	47.34	80.90	6.4130	1422.1	9.42	1.95	1.6460	8.40
270 nM (mr-Oligo)	50.59	81.30	7.0506	1423.7	12.67	2.35	2.2836	10.00
300 nM (mr-Oligo)	54.21	81.70	7.8111	1425.1	16.29	2.75	3.0441	11.40

**Table 9:** COVID-19 test results based on the detection of attributors’ characteristics.

Pathology No.	COVID-19 Test Result Making Condition			Result
01	$\Delta R_{min}^{X}\;\geq \Delta R_{min}^{Y}$ & $\Delta \theta_{SPR}^{X}\geq \Delta \theta_{SPR}^{Y}$	OR	$\Delta T_{max}^{X}\geq \Delta T_{max}^{Y}\;\;$& $\Delta SRF_{freq}^{X}\geq \Delta SRF_{freq}^{Y}$	COVID-19 Positive
02	$\Delta R_{min}^{X}<\Delta R_{min}^{Y}$ & $\Delta \theta_{SPR}^{X}<\Delta \theta_{SPR}^{Y}$		$\Delta T_{max}^{X}<\Delta T_{max}^{Y}\;$& $\Delta SRF_{freq}^{X}<\Delta SRF_{freq}^{Y}$	COVID-19 Negative
03	$\Delta R_{min}^{X}\;\geq \Delta R_{min}^{Y}$ & $\Delta \theta_{SPR}^{X}\leq \Delta \theta_{SPR}^{Y}$		$\Delta T_{max}^{X}\leq \Delta T_{max}^{Y}\;$& $\Delta SRF_{freq}^{X}\geq \Delta SRF_{freq}^{Y}$	Try again
04	$\Delta R_{min}^{X}\;\leq \Delta R_{min}^{Y}$ & $\Delta \theta_{SPR}^{X}\geq \Delta \theta_{SPR}^{Y}$		$\Delta T_{max}^{X}\geq \Delta T_{max}^{Y}\;\;$& $\Delta SRF_{freq}^{X}\leq \Delta SRF_{freq}^{Y}$	Try again

Condition of *X*: *Y* (a) Rapid detection of SARS-CoV-2 Spike RBD: *X* = i and *Y* = SC2R; (b) Rapid detection of SARS-CoV-2 Anti-Spike Protein: *X* = ii and *Y* = IgG; (c) Real-time detection of SARS-CoV-2 RNA Sequence: *X* = iii and *Y* = N.

## Data Availability

Not applicable.

## References

[1] World Health Organization (WHO) (2019). Novel Coronavirus 2019 (COVID-19).

[2] Astuti I. (2020). Ysrafil Severe Acute Respiratory Syndrome Coronavirus 2 (SARS-CoV-2): An overview of viral structure and host response. Diabetes Metab. Syndr..

[3] Wong S.K., Li W., Moore M.J., Choe H., Farzan M. (2004). A 193-Amino Acid Fragment of the SARS Coronavirus S Protein Efficiently Binds Angiotensin-converting Enzyme 2. J. Biol. Chem..

[4] Huang Y., Yang C., Xu X.-F., Xu W., Liu S.-W. (2020). Structural and functional properties of SARS-CoV-2 spike protein: Potential antivirus drug development for COVID-19. Acta Pharmacol. Sin..

[5] Gheblawi M., Wang K., Viveiros A., Nguyen Q., Zhong J.-C., Turner A.J., Raizada M.K., Grant M.B., Oudit G.Y. (2020). Angiotensin-Converting Enzyme 2: SARS-CoV-2 Receptor and Regulator of the Renin-Angiotensin System. Circ. Res..

[6] Knoll M.D., Wonodi C. (2021). Oxford–AstraZeneca COVID-19 vaccine efficacy. Lancet.

[7] Voysey M., Clemens S.A.C., A Madhi S., Weckx L.Y., Folegatti P.M., Aley P.K., Angus B., Baillie V.L., Barnabas S.L., Bhorat Q.E. (2021). Safety and efficacy of the ChAdOx1 nCoV-19 vaccine (AZD1222) against SARS-CoV-2: An interim analysis of four randomised controlled trials in Brazil, South Africa, and the UK. Lancet.

[8] Wise J. (2020). Covid-19: New coronavirus variant is identified in UK. BMJ.

[9] Cui F., Zhou H.S. (2020). Diagnostic methods and potential portable biosensors for coronavirus disease 2019. Biosens. Bioelectron..

[10] Zhao Z., Cui H., Song W., Ru X., Zhou W., Yu X. (2020). A simple magnetic nanoparticles-based viral RNA extraction method for efficient detection of SARS-CoV-2. bioRxiv.

[11] Hou H., Wang T., Zhang B., Luo Y., Mao L., Wang F., Wu S., Sun Z. (2020). Detection of IgM and IgG antibodies in patients with coronavirus disease 2019. Clin. Transl. Immunol..

[12] Li Z., Yi Y., Luo X., Xiong N., Liu Y., Li S., Sun R., Wang Y., Hu B., Chen W. (2020). Development and clinical application of a rapid IgM-IgG combined antibody test for SARS-CoV-2 infection diagnosis. J. Med. Virol..

[13] Lee H.-K., Lee B.-H., Seok S.-H., Baek M.-W., Lee H.-Y., Kim D.-J., Na Y.-R., Noh K.-J., Park S.-H., Kumar D.N. (2010). Production of specific antibodies against SARS-coronavirus nucleocapsid protein without cross reactivity with human coronaviruses 229E and OC43. J. Vet. Sci..

[14] Mak G.C., Cheng P.K., Lau S.S., Wong K.K., Lau C.S., Lam E.T., Chan R.C.W., Tsang D.N.C. (2020). Evaluation of rapid antigen test for detection of SARS-CoV-2 virus. J. Clin. Virol..

[15] Krüttgen A., Cornelissen C.G., Dreher M., Hornef M.W., Imöhl M., Kleines M. (2021). Comparison of the SARS-CoV-2 Rapid antigen test to the real star Sars-CoV-2 RT PCR kit. J. Virol. Methods.

[16] Korevaar D.A., Kootte R.S., Smits L.P., Aardweg J.G.V.D., Bonta P.I., Schinkel J., Vigeveno R.M., Berk I.A.V.D., Scheerder M.J., Lemkes B.A. (2020). Added value of chest computed tomography in suspected COVID-19: An analysis of 239 patients. Eur. Respir. J..

[17] Chen Z.-H., Li Y.-J., Wang X.-J., Ye Y.-F., Wu B.-L., Zhang Y., Xuan W.-L., Bao J.-F., Deng X.-Y. (2020). Chest CT of COVID-19 in patients with a negative first RT-PCR test. Medicine.

[18] Qiu G., Gai Z., Tao Y., Schmitt J., Kullak-Ublick G.A., Wang J. (2020). Dual-Functional Plasmonic Photothermal Biosensors for Highly Accurate Severe Acute Respiratory Syndrome Coronavirus 2 Detection. ACS Nano.

[19] Shrivastav A.M., Cvelbar U., Abdulhalim I. (2021). A comprehensive review on plasmonic-based biosensors used in viral diagnostics. Commun. Biol..

[20] Samson R., Navale G.R., Dharne M.S. (2020). Biosensors: Frontiers in rapid detection of COVID-19. 3 Biotech.

[21] Taha B.A., Al Mashhadany Y., Mokhtar M.H.H., Bin Zan M.S.D., Arsad N. (2020). An Analysis Review of Detection Coronavirus Disease 2019 (COVID-19) Based on Biosensor Application. Sensors.

[22] Hossain B., Islam M., Abdulrazak L.F., Rana M., Akib T.B.A., Hassan M. (2019). Graphene-Coated Optical Fiber SPR Biosensor for BRCA1 and BRCA2 Breast Cancer Biomarker Detection: A Numerical Design-Based Analysis. Photon Sens..

[23] Akib T.B.A., Nazmuschayadat, Hossain B. Superior Performance of Surface Plasmon Resonance Biosensor for Recognizing of DNA Hybridization. Proceedings of the 2019 International Conference on Computer, Communication, Chemical, Materials and Electronic Engineering (IC4ME2).

[24] Peng W., Liu Y., Fang P., Liu X., Gong Z., Wang H., Cheng F. (2014). Compact surface plasmon resonance imaging sensing system based on general optoelectronic components. Opt. Express.

[25] Hossain M.B., Rana M.M. DNA Hybridization Detection Based on Resonance Frequency Readout in Graphene on Au SPR Biosensor. https://www.hindawi.com/journals/js/2016/6070742/.

[26] Tee K.L., Jackson P.J., Scarrott J.M., Jaffe S.R., Johnson A.O., Johari Y., Pohle T.H., Mozzanino T., Price J., Grinham J. (2020). Purification of recombinant SARS-CoV-2 spike, its receptor binding domain, and CR3022 mAb for serological assay. bioRxiv.

[27] Kim D., Lee J., Bal J., Chong C.-K., Lee J., Park H. (2021). Clinical Evaluation of an Immunochromatographic-Based IgM/IgG Antibody Assay (GenBody™ COVI040) for Detection of Antibody Seroconversion in Patients with SARS-CoV-2 Infection. Diagnostics.

[28] Nikolayenko I.V., Galkin O.Y., Grabchenko N.I., Spivak M.Y. (2005). Preparation of highly purified human IgG, IgM, and IgA for immunization and immunoanalysis. Ukr. Bioorganica Acta.

[29] Perchetti G.A., Huang M.-L., Peddu V., Jerome K.R., Greninger A.L. (2020). Stability of SARS-CoV-2 in Phosphate-Buffered Saline for Molecular Detection. J. Clin. Microbiol..

[30] Seo G., Lee G., Kim M.J., Baek S.-H., Choi M., Ku K.B., Lee C.-S., Jun S., Park D., Kim H.G. (2020). Rapid Detection of COVID-19 Causative Virus (SARS-CoV-2) in Human Nasopharyngeal Swab Specimens Using Field-Effect Transistor-Based Biosensor. ACS Nano.

[31] Rahman M., Rana M., Rahman S., Anower M., Mollah A., Paul A.K. (2020). Sensitivity enhancement of SPR biosensors employing heterostructure of PtSe2 and 2D materials. Opt. Mater..

[32] Lee J.-Y., Mai L.-W., Hsu C.-C., Sung Y.-Y. (2013). Enhanced sensitivity to surface plasmon resonance phase in wavelength-modulated heterodyne interferometry. Opt. Commun..

[33] Anower S., Rahman M. (2021). Hybrid Heterostructures for SPR Biosensor. Biosensors.

[34] Guo Y., Singh N.M., Das C.M., Ouyang Q., Kang L., Li K., Coquet P., Yong K.-T. (2020). Two-dimensional PtSe2 Theoretically Enhanced Goos-Hänchen Shift Sensitive Plasmonic Biosensors. Plasmonics.

[35] Jia Y., Li Z., Wang H., Saeed M., Cai H. (2019). Sensitivity Enhancement of a Surface Plasmon Resonance Sensor with Platinum Diselenide. Sensors.

[36] Chabot V., Miron Y., Grandbois M., Charette P.G. (2012). Long range surface plasmon resonance for increased sensitivity in living cell biosensing through greater probing depth. Sens. Actuators B Chem..

[37] Isaacs S., Abdulhalim I. (2015). Long range surface plasmon resonance with ultra-high penetration depth for self-referenced sensing and ultra-low detection limit using diverging beam approach. Appl. Phys. Lett..

[38] Al-Janaby N., Al-Dergazly A. (2020). Fabrication of multi-mode tip fiber sensor based on surface plasmon resonance (SPR). Sustain. Eng. Innov..

[39] Fotovvati B., Namdari N., Dehghanghadikolaei A. (2019). On Coating Techniques for Surface Protection: A Review. J. Manuf. Mater. Process..

[40] Yim C., Lee K., McEvoy N., O’Brien M., Riazimehr S., Berner N.C., Cullen C.P., Kotakoski J., Meyer J.C., Lemme M.C. (2016). High-Performance Hybrid Electronic Devices from Layered PtSe2 Films Grown at Low Temperature. ACS Nano.

[41] O’Brien M., McEvoy N., Motta C., Zheng J.-Y., Berner N.C., Kotakoski J., Elibol K., Pennycook T.J., Meyer J.C., Yim C. (2016). Raman characterization of platinum diselenide thin films. 2D Mater..

[42] Yim C., O’Brien M., McEvoy N., Riazimehr S., Schäfer-Eberwein H., Bablich A., Pawar R., Iannaccone G., Downing C., Fiori G. (2014). Heterojunction Hybrid Devices from Vapor Phase Grown MoS2. Sci. Rep..

[43] Gatensby R., McEvoy N., Lee K., Hallam T., Berner N.C., Rezvani E., Winters S., O’Brien M., Duesberg G.S. (2014). Controlled synthesis of transition metal dichalcogenide thin films for electronic applications. Appl. Surf. Sci..

[44] Wang Y., Li L., Yao W., Song S., Sun J.T., Pan J., Ren X., Li C., Okunishi E., Wang Y.-Q. (2015). Monolayer PtSe2, a New Semiconducting Transition-Metal-Dichalcogenide, Epitaxially Grown by Direct Selenization of Pt. Nano Lett..

[45] Yan M., Wang E., Zhou X., Zhang G., Zhang H., Zhang K., Yao W., Lu N., Yang S., Wu S. (2017). High quality atomically thin PtSe 2 films grown by molecular beam epitaxy. 2D Mater..

[46] Ultra-Fast, Ultra-Sensitive PtSe2 Gas Sensors. https://phys.org/news/2017-01-ultra-fast-ultra-sensitive-ptse2-gas-sensors.html.

[47] Jussila H., Yang H., Granqvist N., Sun Z. (2016). Surface plasmon resonance for characterization of large-area atomic-layer graphene film. Optica.

[48] Ye S., Oh W.-C. (2016). Demonstration of enhanced the photocatalytic effect with PtSe2 and TiO2 treated large area graphene obtained by CVD method. Mater. Sci. Semicond. Process..

[49] Yang X., Peng H., Xie Q., Zhou Y., Liu Z. (2013). Clean and efficient transfer of CVD-grown graphene by electrochemical etching of metal substrate. J. Electroanal. Chem..

[50] Van Ngoc H., Qian Y., Kil Han S., Kang D.J. (2016). PMMA-Etching-Free Transfer of Wafer-scale Chemical Vapor Deposition Two-dimensional Atomic Crystal by a Water Soluble Polyvinyl Alcohol Polymer Method. Sci. Rep..

[51] Sattar S., Schwingenschlögl U. (2017). Electronic Properties of Graphene–PtSe2 Contacts. ACS Appl. Mater. Interfaces.

[52] Gong W., Jiang S., Li Z., Li C., Xu J., Pan J., Huo Y., Man B., Liu A., Zhang C. (2019). Experimental and theoretical investigation for surface plasmon resonance biosensor based on graphene/Au film/D-POF. Opt. Express.

[53] Hossain B., Mehedi I.M., Moznuzzaman M., Abdulrazak L.F., Hossain A. (2019). High performance refractive index SPR sensor modeling employing graphene tri sheets. Results Phys..

[54] Fazio R., Jannelli A., Agreste S. (2018). A Finite Difference Method on Non-Uniform Meshes for Time-Fractional Advection–Diffusion Equations with a Source Term. Appl. Sci..

[55] Menon P.S., Said F.A., Mei G.S., Berhanuddin D.D., Umar A.A., Shaari S., Majlis B.Y. (2018). Urea and creatinine detection on nano-laminated gold thin film using Kretschmann-based surface plasmon resonance biosensor. PLoS ONE.

[56] Hossain B., Akib T.B.A., Abdulrazak L.F., Rana M. (2019). Numerical modeling of graphene-coated fiber optic surface plasmon resonance biosensor for BRCA1 and BRCA2 genetic breast cancer detection. Opt. Eng..

[57] Rahman M.S., Rikta K.A., Bin Bashar L., Anower M. (2018). Numerical analysis of graphene coated surface plasmon resonance biosensors for biomedical applications. Optica.

[58] Lin C., Chen S. (2019). Design of high-performance Au-Ag-dielectric-graphene based surface plasmon resonance biosensors using genetic algorithm. J. Appl. Phys..

[59] Rahman M.S., Hossain B., Rana M. Sensitivity enhancement of porous silicon based SPR sensor using graphene-M0S2 hybrid structure. Proceedings of the 2nd International Conference on Electrical, Computer & Telecommunication Engineering (ICECTE).

[60] Islam M., Islam M., Shimul Y.C., Rahman A., Ruhe A.A., Hassan M., Hossain B. (2019). FDTD Analysis Fiber Optic SPR Biosensor for DNA Hybridization: A Numerical Demonstration with Graphene. J. Mater. Appl..

[61] De Moivre’s Formula. https://en.wikipedia.org/w/index.php?title=De_Moivre%27s_formula&oldid=1006955752.

[62] Cennamo N., Massarotti D., Galatus R.V., Conte L., Zeni L. (2013). Performance Comparison of Two Sensors Based on Surface Plasmon Resonance in a Plastic Optical Fiber. Sensors.

[63] Meshginqalam B., Barvestani J. (2018). Performance Enhancement of SPR Biosensor Based on Phosphorene and Transition Metal Dichalcogenides for Sensing DNA Hybridization. IEEE Sens. J..

[64] Das C.M., Guo Y., Kang L., Ho H., Yong K. (2020). Investigation of Plasmonic Detection of Human Respiratory Virus. Adv. Theory Simulations.

[65] Das C.M., Guo Y., Yang G., Kang L., Xu G., Ho H., Yong K. (2020). Gold Nanorod Assisted Enhanced Plasmonic Detection Scheme of COVID-19 SARS-CoV-2 Spike Protein. Adv. Theory Simul..

[66] Ouyang Q., Zeng S., Jiang L., Hong L., Xu G., Dinh X.Q., Qian J., He S., Qu J., Coquet P. (2016). Sensitivity Enhancement of Transition Metal Dichalcogenides/Silicon Nanostructure-based Surface Plasmon Resonance Biosensor. Sci. Rep..

[67] Xie J., Zhang D., Yan X.-Q., Ren M., Zhao X., Liu F., Sun R., Li X., Li Z., Chen S. (2019). Optical properties of chemical vapor deposition-grown PtSe 2 characterized by spectroscopic ellipsometry. 2D Mater..

[68] Bruna M., Borini S. (2009). Optical constants of graphene layers in the visible range. Appl. Phys. Lett..

[69] Fu H., Zhang S., Chen H., Weng J. (2015). Graphene Enhances the Sensitivity of Fiber-Optic Surface Plasmon Resonance Biosensor. IEEE Sens. J..

[70] Shushama K.N., Rana M., Inum R., Hossain B. (2017). Graphene coated fiber optic surface plasmon resonance biosensor for the DNA hybridization detection: Simulation analysis. Opt. Commun..

[71] Hossain B., Rana M. (2016). Graphene Coated High Sensitive Surface Plasmon Resonance Biosensor for Sensing DNA Hybridization. Sens. Lett..

[72] Patching S.G. (2014). Surface plasmon resonance spectroscopy for characterisation of membrane protein–ligand interactions and its potential for drug discovery. Biochim. Biophys. Acta Biomembr..

[73] Brogioni B., Berti F. (2014). Surface plasmon resonance for the characterization of bacterial polysaccharide antigens: A review. MedChemComm.

[74] Schasfoort R.B.M. (2017). Chapter 1. Introduction to Surface Plasmon Resonance. Handbook of Surface Plasmon Resonance.

[75] Zhang J., Zhang L., Xu W. (2012). Surface plasmon polaritons: Physics and applications. J. Phys. D Appl. Phys..

[76] Forssén P., Samuelsson J., Lacki K., Fornstedt T. (2020). Advanced Analysis of Biosensor Data for SARS-CoV-2 RBD and ACE2 Interactions. Anal. Chem..

[77] Lan J., Ge J., Yu J., Shan S., Zhou H., Fan S., Zhang Q., Shi X., Wang Q., Zhang L. (2020). Structure of the SARS-CoV-2 spike receptor-binding domain bound to the ACE2 receptor. Nature.

[78] Forssén P., Multia E., Samuelsson J., Andersson M., Aastrup T., Altun S., Wallinder D., Wallbing L., Liangsupree T., Riekkola M.-L. (2018). Reliable Strategy for Analysis of Complex Biosensor Data. Anal. Chem..

[79] Tian X., Li C., Huang A., Xia S., Lu S., Shi Z., Lu L., Jiang S., Yang Z., Wu Y. (2020). Potent binding of 2019 novel coronavirus spike protein by a SARS coronavirus-specific human monoclonal antibody. Emerg. Microbes Infect..

[80] Yu F., Xiang R., Deng X., Wang L., Yu Z., Tian S., Liang R., Li Y., Ying T., Jiang S. (2020). Receptor-binding domain-specific human neutralizing monoclonal antibodies against SARS-CoV and SARS-CoV-2. Signal Transduct. Target. Ther..

[81] Lv Z., Deng Y.-Q., Ye Q., Cao L., Sun C.-Y., Fan C., Huang W., Sun S., Sun Y., Zhu L. (2020). Structural basis for neutralization of SARS-CoV-2 and SARS-CoV by a potent therapeutic antibody. Science.

[82] Wee S.K., Sivalingam S.P., Yap E.P.H. (2020). Rapid Direct Nucleic Acid Amplification Test without RNA Extraction for SARS-CoV-2 Using a Portable PCR Thermocycler. Genes.

[83] Xu Y., Li X., Zhu B., Liang H., Fang C., Gong Y., Guo Q., Sun X., Zhao D., Shen J. (2020). Characteristics of pediatric SARS-CoV-2 infection and potential evidence for persistent fecal viral shedding. Nat. Med..

[84] Li D., Zhang J., Li J. (2020). Primer design for quantitative real-time PCR for the emerging Coronavirus SARS-CoV-2. Theranostics.

[85] Long Q.-X., Liu B.-Z., Deng H.-J., Wu G.-C., Deng K., Chen Y.-K., Liao P., Qiu J.-F., Lin Y., Cai X.-F. (2020). Antibody responses to SARS-CoV-2 in patients with COVID-19. Nat. Med..

[86] Denning D.W., Kilcoyne A., Ucer C. (2020). Non-infectious status indicated by detectable IgG antibody to SARS-CoV-2. Br. Dent. J..

